# Immune Cell Landscape of Patients With Diabetic Macular Edema by Single-Cell RNA Analysis

**DOI:** 10.3389/fphar.2021.754933

**Published:** 2021-09-14

**Authors:** Pengjuan Ma, Ping Zhang, Shuxia Chen, Wen Shi, Jinguo Ye, Shida Chen, Rong Ju, Bingqian Liu, Yingfeng Zheng, Yizhi Liu

**Affiliations:** ^1^ State Key Laboratory of Ophthalmology, Zhongshan Ophthalmic Center, Sun Yat-sen University, Guangzhou, China; ^2^ Guangzhou Regenerative Medicine and Health Guangdong Laboratory, Guangzhou, China; ^3^ Research Unit of Ocular Development and Regeneration, Chinese Academy of Medical Sciences, Guangzhou, China

**Keywords:** single-cell sequencing, peripheral blood mononuclear cells, monocytes, DME, chronic vascular inflammation

## Abstract

**Purpose:** We performed single-cell RNA sequencing (scRNA-seq), an unbiased and high-throughput single cell technology, to determine phenotype and function of peripheral immune cells in patients with diabetic macular edema (DME).

**Methods:** Peripheral blood mononuclear cells (PBMCs) were isolated from DME patients and healthy controls (HC). The single-cell samples were loaded on the Chromium platform (10x Genomics) for sequencing. R package Seurat v3 was used for data normalizing, clustering, dimensionality reduction, differential expression analysis, and visualization.

**Results:** We constructed a single-cell RNA atlas comprising 57,650 PBMCs (24,919 HC, 32,731 DME). We divided all immune cells into five major immune cell lineages, including monocytes (MC), T cells (TC), NK cells (NK), B cells (BC), and dendritic cells (DC). Our differential expression gene (DEG) analysis showed that MC was enriched of genes participating in the cytokine pathway and inflammation activation. We further subdivided MC into five subsets: resting CD14^++^ MC, proinflammatory CD14^++^ MC, intermediate MC, resting CD16^++^ MC and pro-inflammatory CD16^++^ MC. Remarkably, we revealed that the proinflammatory CD14^++^ monocytes predominated in promoting inflammation, mainly by increasingly production of inflammatory cytokines (*TNF*, *IL1B*, and *NFKBIA*) and chemokines (*CCL3*, *CCL3L1*, *CCL4L2*, *CXCL2*, and *CXCL8*). Gene Ontology (GO) and pathway analysis of the DEGs demonstrated that the proinflammatory CD14^++^ monocytes, especially in DME patients, upregulated inflammatory pathways including tumor necrosis factor-mediated signaling pathway, I-kappaB kinase/NF-kappaB signaling, and toll-like receptor signaling pathway.

**Conclusion:** In this study, we construct the first immune landscape of DME patients with T2D and confirmed innate immune dysregulation in peripheral blood based on an unbiased scRNA-seq approach. And these results demonstrate potential target cell population for anti-inflammation treatments.

## Introduction

Diabetic retinopathy (DR) is a significant microvascular complication of diabetes, dividing into nonproliferative diabetic retinopathy (NPDR) and proliferative diabetic retinopathy (PDR) clinically based on the proliferative status of the retinal vasculature (Cheung et al., 2010). Diabetic macular edema (DME) is a significant complication of DR, mainly caused by the breakdown of the blood-retinal barrier (BRB) and leaking microaneurysms (Xu and Le, 2011). DME can occur at any stage in the pathogenesis of DR and lead to severe vision loss in diabetic patients (Das et al., 2015).

Recent studies suggest that DR is a chronic low-grade inflammatory disease (Donath and Shoelson, 2011; Tang and Kern, 2011). Inflammation plays a critical role in diabetic retinopathy and contributes to vascular permeability and edema by releasing inflammatory cytokines, leukocyte activation, and leukostasis (Miyamoto et al., 1999; Chibber et al., 2000; Noda et al., 2012; Mesquida et al., 2019). Increasing evidence indicates that cytokines such as interleukin-1β (IL-1β), tumor necrosis factor α (TNF-α), IL-6, and IL-8 were significantly upregulated in vitreous and serum of DR patients, resulting in a persistent chronic inflammation state in the retina (Ben-Mahmud et al., 2004; Demircan et al., 2006; Boss et al., 2017; Feng et al., 2018; Khaloo et al., 2020). Innate immune cells, especially monocytes, are reported to play a pivotal role in promoting the pathogenesis of DR (Van Hove et al., 2020; Wan et al., 2020). In peripheral blood of diabetic patients with microvascular or macrovascular complications, CD45^+^CD14^+^ classical monocytes were increased, but CD16^+^ nonclassical monocytes were decreased, compared with patients without complications (Min et al., 2012). Serra et al. showed that circulating CD11b monocytes from diabetic mice were preferentially trapped in retinal microvascular bed and may lead to diabetic retinal vasculopathy by expressing higher levels of chemokine receptor CCR5(Serra et al., 2012). Recently, neutrophils were identified to promote microvascular occlusions and small-vessel vasculitis by producing neutrophil extracellular traps in PDR (Binet et al., 2020). These studies indicate that immune cells may play essential roles via attaching to vascular endothelium and cause retinal vasculopathy. One single-cell RNA sequencing study has established a high-resolution transcriptome landscape of blood immune cell subsets in T1D children and revealed a high level of IL-32 produced mainly by activated T cells and NK cells could be an early indicator for T1D (Kallionpaa et al., 2019).

Despite these studies, it is still unclear whether chronic vascular inflammation accelerates BRB breakdown and fluid accumulation in DME patients with T2D. Furthermore, little is known regarding the phenotypic and functional diversity of different immune cell types in DME. In addition, their contribution to vascular inflammation remains to be fully elucidated. Thus, defining key cell subsets and their states in DME is crucial in acquiring critical insights into the immune mechanisms and developing new therapeutic strategies for DME.

Here, to clarify the phenotype and function of peripheral immune cells in DME, we utilized single-cell RNA sequencing (scRNA-seq) to comprehensively characterize the transcriptional heterogeneity of PBMCs from healthy individuals and DME patients. Our study depicted a landscape of blood immune cell subsets, including monocytes, dendritic cells, NK, T, and B cells, and characterized their gene expression programs.

## Materials and Methods

### Human Subjects

Four DME patients and four healthy individuals were enrolled at the Zhongshan Ophthalmic Center, Guangzhou, China. All patients were diagnosed with type 2 diabetes with diabetic retinopathy determined by fluorescein angiography and comprehensive ophthalmologic examinations. The clinical-stage of diabetic retinopathy was classified according to the International Clinical Diabetic Retinopathy (Wilkinson et al., 2003). The characteristic of the patients is shown in Supplemental Table S1. We selected DME patients with a central macular thickness of 300 μm or more evaluated by optical coherence tomography (OCT). Individuals with autoimmune disease, cancer, cardiovascular diseases, and other eye diseases (such as age-related macular degeneration, cystoid macular edema of other origins, uveitis) were excluded to avoid confusion with other systemic diseases. Written informed consent was obtained from all patients after explaining the purpose and procedures to be used. The study was approved by the Ethics Committee of Zhongshan Ophthalmic Center, China.

### Isolation of Peripheral Blood Mononuclear Cells (PBMCs) for scRNA-Seq

Blood samples from healthy individuals and patients were processed within 2 h after collection and diluted 1:1 with phosphate-buffered saline (PBS, Gibco, C10010500BT). Then, the diluted samples were layered onto the Ficoll-Paque PLUS (GE Healthcare Life Sciences, 17-1440-03) in the centrifuge tubes and centrifuged at 400 g for 30 min at 18–20°C. The PBMCs layer was collected and washed twice in PBS and identified the viability and quantity of single cells using Trypan blue. If the cell survival rate exceeded 90%, PBMC samples were used for the following scRNA-seq experiment.

### scRNA-Seq

The single-cell samples were loaded on the Chromium platform (10x Genomics) for library preparation, and the barcoded scRNA-seq libraries were constructed using the Chromium Single Cell 5′ Reagent kit (10x Genomics) and following the manufacturer’s instructions. In brief, single-cell gel beads in emulsions (GEMs) were generated, and reverse transcription (RT) was performed to produce 10x barcoded, full-length cDNA from polyadenylated mRNA. Then, the 10x barcoded cDNA was amplified via PCR, followed by enzymatic fragmentation, end repair, A-tailing, adaptor ligation, and sample index PCR. After the library preparation was completed, the next-generation sequencing was performed on the 10x Genomics Chromium Illumina NovaSeq6000 platform according to Illumina standard procedures. The quality of the libraries was checked using the FastQC software.

### ScRNA-Seq Data Alignment and Quality Control

The raw sequencing data of patients and healthy controls were demultiplexed by CellRanger Software (version 3.1.0) and aligned to the GRCh38 human reference genome with default parameters. The CellRanger count function was used to generate single-cell feature counts for a single library, and the CellRanger aggr function was used to aggregate gene counts of all patients and healthy controls. The single-cell expression matrix was further analyzed by Seurat (V3) according to the tutorial at https://satijalab.org/seurat/site. For quality control, high-quality cells were retained following the criteria: 1) gene number was between 200 and 3,000; 2) the percentage of mitochondrial RNA was <8% per cell. Low-quality cells with high HBB and HBA1 expression levels were also filtered, which identified as the RBC-contaminated cell population. After quality control, 57,650 cells (24,919 HC and 32,731 DME) were left for the following analysis. Mitochondria (M.T.) and ribosomes (RPL and RPS) genes were also eliminated in downstream analysis.

### Dimensionality Reduction and Clustering Analysis

Data normalization, scaling, clustering, dimensionality reduction, differential expression analysis, and visualization were processed using the R package Seurat. The global-scaling normalization method “LogNormalize” was employed to normalize the feature expression measurements for each cell. Highly variable features were identified by the FindVariableFeatures function, and data was scaled by the ScaleData function. Moreover, the R package harmony was used to remove batch effects to create a corrected expression matrix for further analysis. Next, dimensionality reduction was performed using principal component analysis (PCA), and cell clusters were visualized with the t-distributed stochastic neighbor embedding (t-SNE) algorithm.

### ScRNA-Seq Differential Expression Analysis

Seurat package FindMarkers function with default parameters was used to perform differential gene expression analysis between the control and disease groups of the same cell type. The Wilcoxon rank-sum test within the FindAllMarkers function was used to analyze all single-cell differential gene expression for identified cell subsets. The marker genes for each cluster were detected by comparing them against all other cells in the experiment. The upregulated and downregulated differentiated expressed genes among different comparisons were shown by the volcano plots. In addition, the Venn diagram was used to show the overlap of differentiated expressed genes among different cell clusters.

### GO and Pathway Enrichment Analysis

The detected differentiated expressed genes were further used to perform Gene Ontology, gene-set enrichment analysis, and KEGG pathway analysis using Metascape webtool (www.metascape.org) (Zhou et al., 2019).

### Transcription Factor Module Analysis

The gene regulatory network of monocytes was constructed by SCENIC, a computational method to predict critical regulators and identify cell state from single-cell RNA -seq data. The R package GENIE3 was first used to generate co-expression gene regulatory networks (GRN), and the co-expression data was then subjected to *cis*-regulatory motif analysis using the R package RcisTarget. Furthermore, the AUCell algorithm was used to score the activity of significant regulons enriched in different clusters.

### Cell-Cell Communication Analysis

The cell-cell communication networks between monocytes and other cell clusters were performed using CellphoneDB statistical analysis, a computational approach predicting cell-cell interactions by ligand-receptor interactions analysis. The ligand-receptor interactions calculated by CellphoneDB were based on the expression of a ligand by one cell cluster and a receptor by another cell cluster. Using this method, we compared the enriched ligand-receptor interactions in DME with HC and NPDR. Furthermore, the dot plots generated by R package ggplot2 were used to visualize the top significant interactions in DME.

### Statistical Analysis

The Wilcoxon rank-sum test was used to identify the DEGs and compare the differences in the expression of genes of interest between the HC and DME groups. The hypergeometric test and the Benjamini- Hochberg *p* value were used in Metascape to identify the ontology terms. Furthermore, we used Wilcoxon rank-sum test to assess the significance of the number differences of MC subsets between the HC and DME group.

## Results

### Study Design and Analysis for Single-Cell Immunophenotyping in DME Patients

**FIGURE 1:**
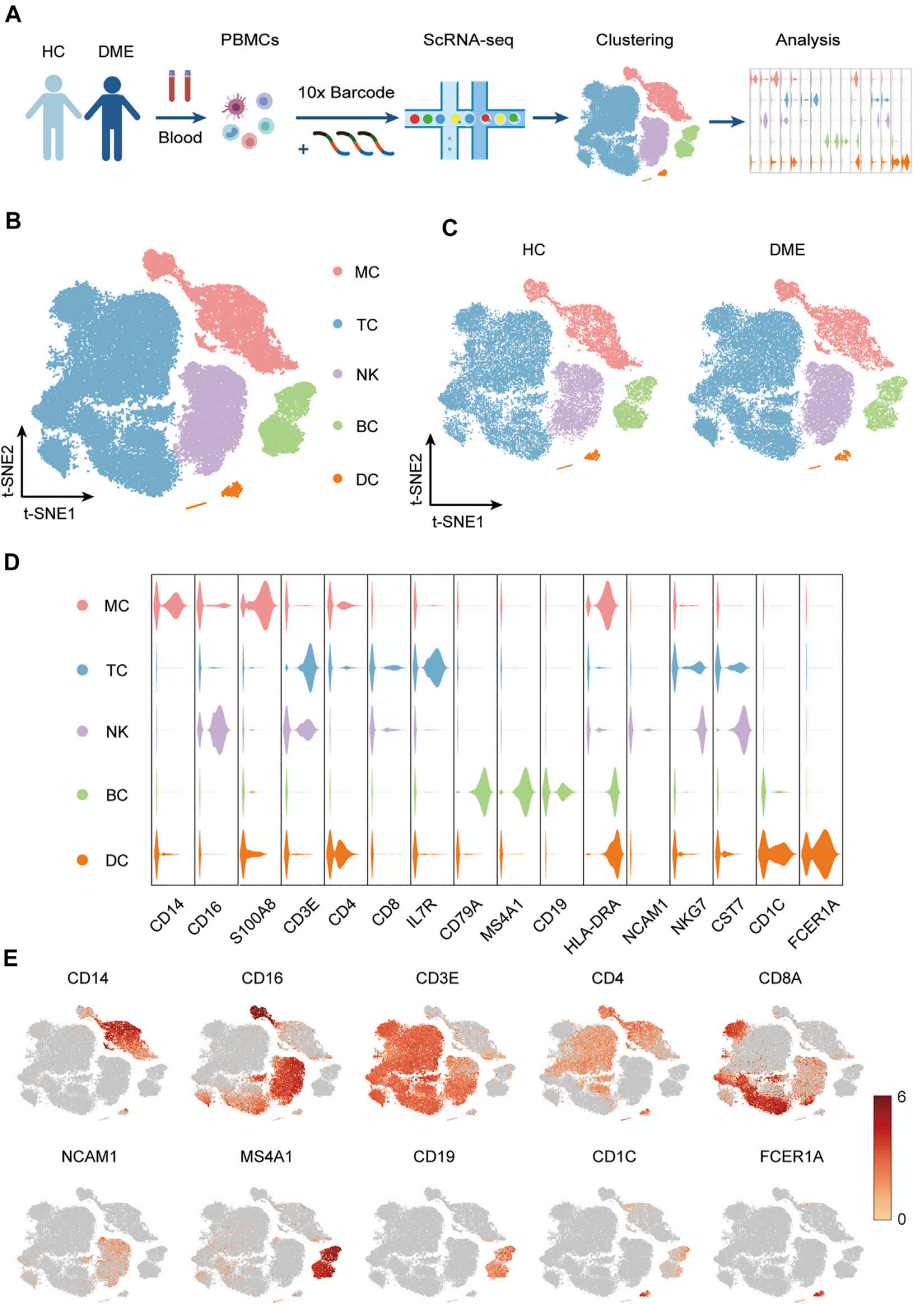
Experimental approach and characterization immune cell clusters from scRNA-seq data. **(A)** Experimental outline showing PBMC collection and scRNA-seq data analysis. **(B)** T-sne plot of major immune cell clusters in PBMCs. Cell types are labeled with colors as indicated. Monocytes (MC); T cells (TC); NK cells (NK); B cells (BC) and dendritic cells (DC). **(C)** T-sne plot of cell clusters in HC and DME respectively. Cell types are labeled with colors as indicated. HC: health control; DME: diabetic macular edema. **(D)** Violin plot of major immune cell clusters in PBMCs, clustered by their relative expression of the cell type-specific markers. Monocytes (MC); T cells (TC); NK cells (NK); B cells (BC) and dendritic cells (DC). **(E)** The feature plot showing the expression of representative markers in each cluster.

To profile the peripheral immune microenvironment of DME, we performed single-cell RNA sequencing (scRNA-seq) to investigate PBMCs from four prospectively enrolled DME patients with T2D and four healthy donors as controls (Figure 1A). Single-cell suspensions of PBMCs were collected and converted to barcoded scRNA-seq libraries using 10X Genomics. CellRanger software was used for the initial processing of the sequencing data. Quality metrics included the number of unique molecular identifiers (UMI), genes detected per cell, and reads aligned to the human genome. Miscellaneous cells with high HBB and HBA1 expression levels were filtered, which identified as the RBC-contaminated cell population. After quality control, a total of 57,650 cells (24,919 HC and 32,731 DME) were used for downstream analysis.

Based on the expression of canonical markers in each cluster, we divided all immune cells into five major immune cell lineages, including monocytes (MC), T cells (TC), NK cells (NK), B cells (BC), and dendritic cells (DC). Then we generated two-dimensional visualization of the high-throughput sequencing data using t-distributed stochastic neighbor embedding (t-SNE), an unbiased dimensionality reduction algorithm (Figure 1B). We demonstrated that the residual batch effect was removed, and the scRNA-seq data across different groups showed consistent repeatability after gene expression normalization (Figure 1C). The violin plots indicated expression levels, and the t-SNE maps confirmed the relative distribution of cell type-specific marker genes across all clusters (Figures 1D,E). These plots showed that each cluster was identified by their unique signature genes: *CD14* and *CD16* (MC marker), *CD3E*, *CD4* and *CD8A* (TC marker), *NCAM1* (NK marker), *MS4A1* and *CD19* (BC marker), *CD1C* and *FCER1A* (DC marker) (Figures 1D,E).

### Proinflammatory Phenotype Mediated by Monocytes in DME Patients

**FIGURE 2:**
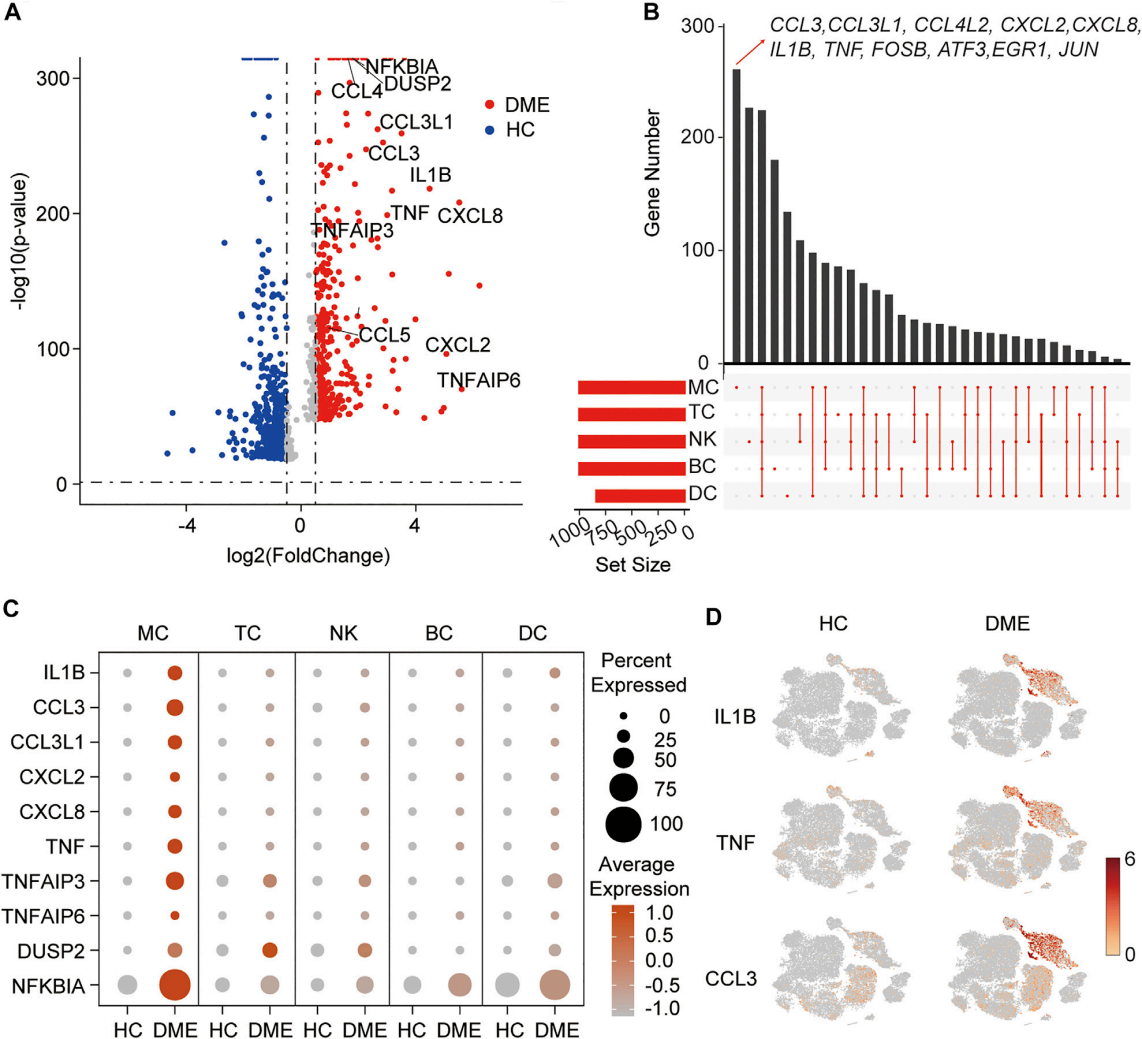
A proinflammatory phenotype mediated by MC in DME patients. **(A)** Volcano plot showing up-regulated and down-regulated DEGs of all immune cells in DME compared to in HC (DME: red; HC: blue). DEG: differential expression gene. **(B)** Venn plot showing unique upregulated genes expressed in MC, DC, TC, NK and BC from DME compared to the ones from HC. **(C)** The dot plot showing the expression of inflammatory genes in the five major cell clusters from the HC and DME group. **(D)** The feature plot showing the expression of representative inflammatory genes, *ILB*, *TNF*, and *CCL3*, in each cluster from the HC and DME group.

It has been demonstrated that immune cells, such as T cells, NK cells, and monocytes, play different roles in neovascularization and vascular permeability in diabetic retinopathy (Kallionpaa et al., 2019; Wan et al., 2020). However, the predominant immune cell populations contributing to macular edema in DME remain unknown. Firstly, to understand the transcriptional changes in the immune cells, we conducted a comparative analysis of differential expression genes (DEGs) between HC and DME patients. The volcano plot revealed that inflammatory-related genes (*TNF, TNFAIP6, IL1B, NFKBIA,* and *DUSP2*), chemokines (*CCL3, CXCL2,* and *CXCL8*) were all expressed at high levels in DME patients compared to HC (Figure 2A). These highly expressed inflammation-associated genes implied that the immune cells in the blood of DME patients were in a proinflammatory state, which may contribute to vascular endothelial cell damage and retinopathy.

To further investigate transcriptional heterogeneity of immune cell signatures, we analyzed DEGs of each cluster in DME patients compared to HC, and we showed unique and shared upregulated expression genes of MC, TC, NK, BC, and DC in DME using the venn plot (Figure 2B). Specific upregulated DEGs of the five immune cell subsets were different in numbers and presented distinct biological functions. Remarkably, we found MC contained the highest number of specific upregulated genes, followed by NK (Figure 2B). Interestingly, upregulated DEGs of DME, including inflammatory genes (for example, *CCL3*, *IL1B*, and *TNF*) and transcriptional factors (for example, *FOSB* and *JUN*) found in the volcano plot were significantly differentially expressed by MC only (Figures 2A,B), suggesting that MC may mediate the expression changes to activate the inflammatory response. Except for MC, other clusters displayed a resting state with low numbers of proinflammatory genes (Figure 2B).

In order to systematically show the differences in the DEGs of immune cell subsets, we compared the expression level of the top inflammatory genes among the five clusters in HC and DME, respectively. The MC had the most robust inflammatory signature with high levels of cytokine genes expression among five immune cell subsets (Figure 2C). Our comparison between the two groups also suggested the considerable accumulation of increased cytokine activity in DME patients (Figure 2C). The t-SNE maps further confirmed that distribution of upregulated inflammatory genes, such as *IL1B*, *TNF*, and *CCL3*, concentrated in MC of DME patients especially (Figure 2D).

### A Proinflammatory Monocyte Subset Predominated in the Pathological Process of DME

**FIGURE 3:**
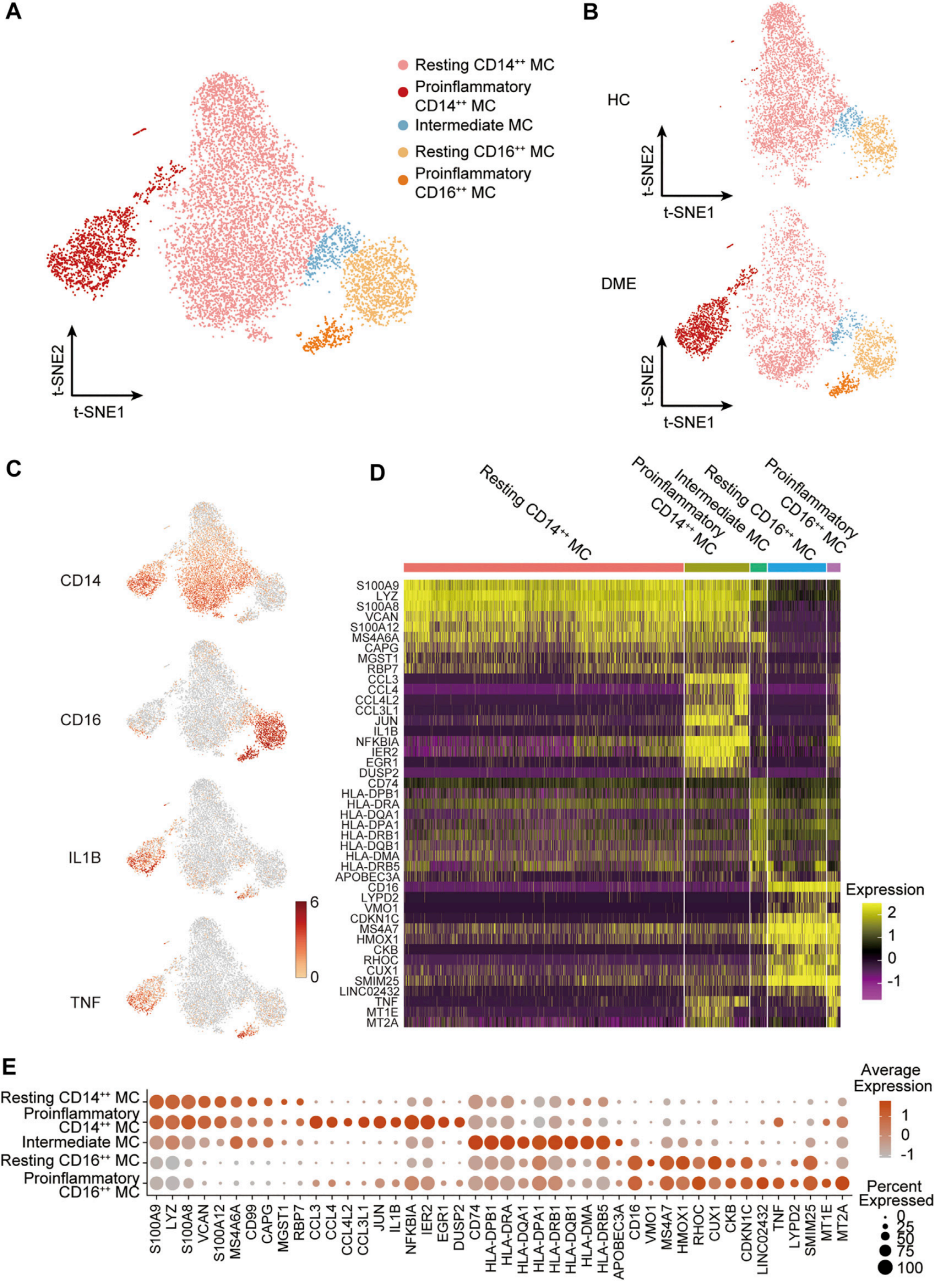
MC subset classification and characterization in DME patients. **(A)** T-sne plot of major MC subsets. Cell subtypes are labeled with colors as indicated. **(B)** T-sne plot of major MC subsets in HC and DME, respectively. Cell subtypes are labeled with colors as indicated. **(C)** The feature plot showing the expression of canonical lineage markers in all clusters. **(D)** The heatmap showing expression of top 10 marker genes in all MC subsets. **(E)** The dot plot showing expression of top 10 marker genes in all MC subsets.

**FIGURE 4:**
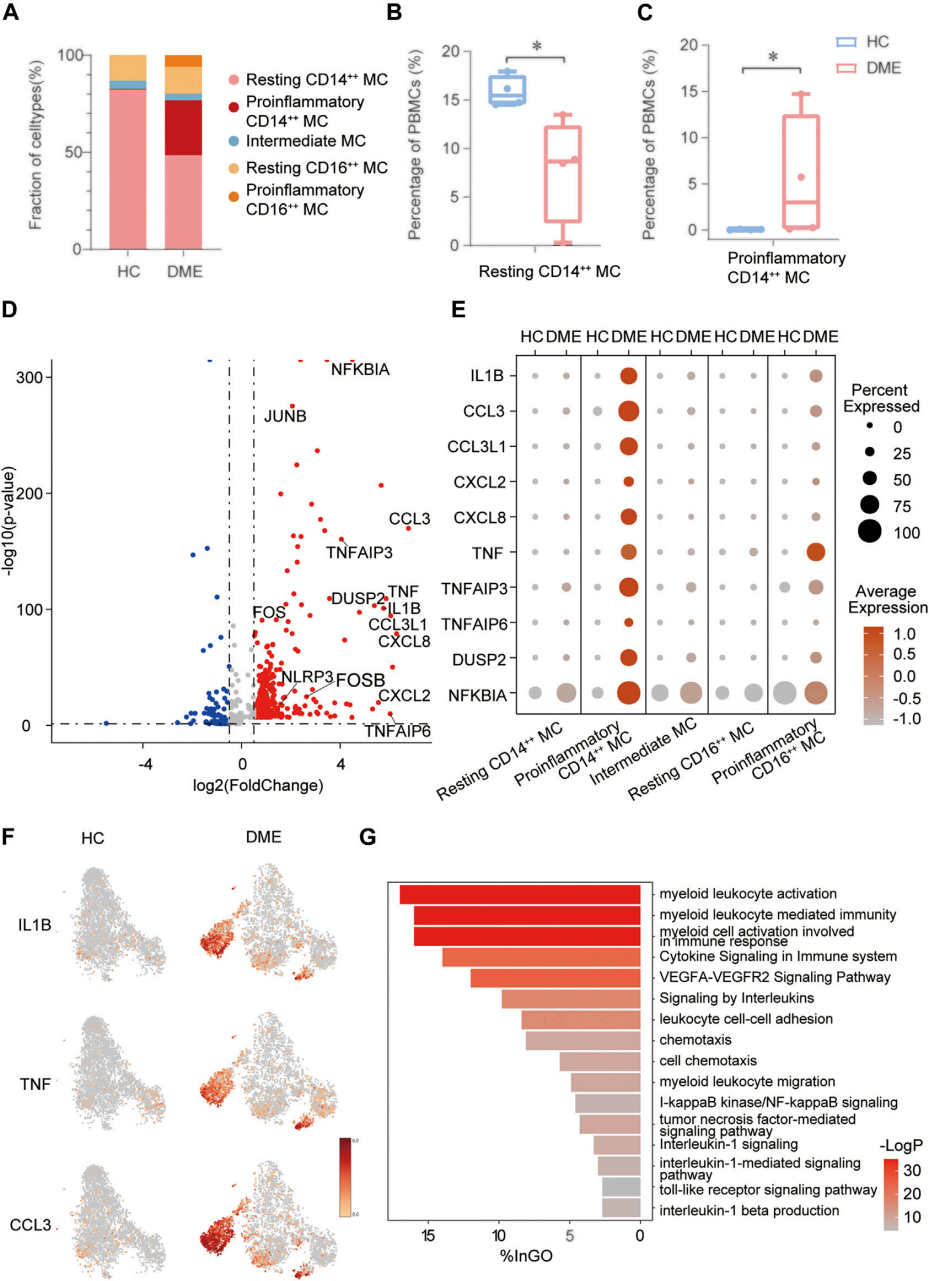
The inflammatory genes and signaling pathways enriched in proinflammatory CD14^++^ MC. **(A)** Comparation of relative fractions of MC subsets from the HC and DME group. **(B)** Quantification of resting CD14^++^ MC in PBMCs from the DME group compared to the HC group. (n = 4, *: *p* value <0.05). **(C)** Quantification of proinflammatory CD14^++^ MC in PBMCs from the DME group compared to the HC group. (n = 4, *: *p* value <0.05). **(D)** Volcano plot showing up-regulated and down-regulated DEGs of MC in DME compared to in HC (DME: red; HC: blue). **(E)** The dot plot showing the expression of inflammatory genes in the five MC subsets from the HC and DME group. **(F)** The feature plot showing the expression of inflammatory markers from the HC and DME group. **(G)** The bar plot showing the signaling pathways enriched in proinflammatory CD14^++^ MC by Gene Ontology (GO) analysis.

The analysis above demonstrated that MC was the main proinflammatory cell in DME. Based on the relative expression of *CD14* and *CD16*, monocytes can be traditionally subclassified as classical (CD14^++^ CD16^−^), nonclassical (CD14^dim^ CD16^++^), and intermediate monocytes (CD14^++^ CD16^+^) (Ziegler-Heitbrock et al., 2010). To further understand the heterogeneity of MC in DME patients, we re-clustered all the MC and conducted precise cell classification (Figure 3A). We observed distinct distributions of MC subsets in HC and DME on the t-SNE maps (Figure 3B). Based on the expression level of canonical lineage markers (*CD14* and *CD16*) and inflammatory-related markers (*IL1B* and *TNF*) (Figure 3C), we classified five monocyte subsets and described each subset by the top 10 markers (Figure 3D). Here, we discovered that the CD14^++^ MC consisted of two clusters (Figure 3D). One presented high *CD14* gene expression and low inflammatory gene expression signature, namely resting CD14^++^ MC. Another cluster called proinflammatory CD14^++^ MC exhibited high *CD14* and inflammatory gene expression (Figure 3D), indicating that this subpopulation may be associated with pathogenic processes through inflammation activation. Furthermore, the CD16^++^ MC can also be re-clustered into two subsets. So, we subdivided MC into five subsets: resting CD14^++^ MC, proinflammatory CD14^++^ MC, intermediate MC, resting CD16^++^ MC and proinflammatory CD16^++^ MC, based on the expression of canonical monocyte marker genes and proinflammatory genes (Figure 3D). From the heatmap and dot-plot of the five MC subsets, we found that both the CD14^++^ MCs expressed not only recognized markers (*S100A9*, *LYZ*, *S100A8*, *VCAN*, and *S100A12*) but also newly identified markers (*MS4A6A*, *CAPG*, *MGST1*, and *RBP7*) (Figures 3D,E). The proinflammatory CD14^++^ MC highly expressed distinguishing biomarkers, including inflammatory markers (*CCL3*, *CCL4*, *CCL4L2*, *CCL3L1*, *IL1B*, *NFKBIA*, and *TNF*) and typical transcription factors (*IER2* and *EGR1*) (Figures 3D,E). The intermediate MC with high CD14 and moderate CD16 expression was at the connection of the CD14^++^ MCs, and CD16^++^ MCs showed in the t-SNE plot (Figure 3A). HLA-related genes, including *HLA-DPB1, HLA-DRA,* and *HLA-DQA1,* were upregulated in this subset, suggesting an increased antigen processing and presentation (Figures 3D,E). The two CD16^++^ MCs represented nonclassical monocytes with high expression of *CD16* and other unique signature makers identified by our scRNA-seq data, such as *LYPD2, VM O 1, CDKN1C, MS4A7,* and *HMOX1* (Figures 3D,E). Inflammatory markers (*TNF*, *IL1B,* and *NFKBIA*) were highly expressed in the proinflammatory CD16^++^ MC compared to the resting CD16^++^ MC (Figures 3D,E). Among all the MC subsets, the proinflammatory CD14^++^ MC expressed the highest level of inflammatory genes (Figures 3D,E). We also observed that the composition of cell subsets in MC differed largely between the HC and DME groups, and the fraction of proinflammatory CD14^++^ MC was remarkably elevated in DME patients (Figure 4A). Furthermore, the frequency of proinflammatory CD14^++^ MC in all PBMCs was significantly increased in DME compared to HC (HC vs DME; *p* < 0.05; Figure 4C), while resting CD14^++^ MC was significantly decreased (HC vs DME; *p* < 0.05; Figure 4B). These results suggested that CD14^++^ MC in DME patients turned from a resting state into a pro-inflammatory state.

### The Inflammatory Genes and Signaling Pathways Were Enriched in the Proinflammatory CD14^++^ MC

In order to delineate how MC changed between HC and DME, we first compared the unique DEGs of each group. The MC in DME was uniquely characterized by the upregulation of inflammatory genes, including inflammatory cytokines (*TNF*, *IL1B*, *NFKBIA*, *DUSP2*, *NLRP3*, and *TNFAIP6*), chemokines (*CCL3*, *CCL3L1*, *CCL4L2*, *CXCL2*, and *CXCL8*), and transcriptional factors (*FOS*, *FOSB*, and *JUNB*) (Figure 4D). The most distinct transcriptional differences of the proinflammatory CD14^++^ MC compared to other MC subsets were the higher levels of inflammatory genes, consistent with a proinflammatory phenotype (Figure 4E). Furthermore, the dot plot revealed DME patients owned the most activated MC, while MC in H.C. presented a resting state (Figure 4E). We further identified that distribution of upregulated inflammatory genes, such as *IL1B*, *TNF*, and *CCL3*, concentrated in the proinflammatory CD14^++^ MC of DME patients especially confirmed by the t-SNE maps (Figure 4F). These analysis data further confirmed that MC, especially the proinflammatory CD14^++^ MC, played an essential role in the pathogenesis of DME by inflammation activation.

We identified five distinct MC subsets and demonstrated the most activated and proinflammatory MC by transcriptional analysis. To further investigate functional heterogeneity of the proinflammatory CD14^++^ MC in DME, we analyzed signaling pathways of this subset by Gene Ontology (G.O.) and pathway enrichment analysis using the upregulated DEGs in DME compared to HC (Figure 4G). Consistent with the high levels of inflammatory genes, signaling pathways related to monocytes activation and inflammatory response were enriched in the proinflammatory CD14^++^ MC of DME (Figure 4G). When activated, circulating monocytes were recruited to the sites of inflammation and initiated immune responses in the pathology of many diseases (Shi and Pamer, 2011). As for DME, the proinflammatory CD14^++^ MC was characterized by highly upregulated myeloid leukocyte activation, migration, and chemotaxis pathways (Figure 4G). We found the VEGFA-VEGFR2 pathway, known to promote neovascularization and BRB breakdown, was also significantly up-regulated (Figure 4G). The abundance of inflammatory cytokine-related pathways, such as cytokine signaling in the immune system, signaling by interleukins, and interleukin-1 beta production, revealed that the proinflammatory CD14^++^ MC initiated inflammatory responses mainly by cytokine production, especially *IL1B* (Figure 4G). This MC subset also enhanced cell adhesion pathways (Figure 4G), indicating that monocytes may interact with other immune cells and retinal vascular endothelial cells. Common upregulated inflammatory pathways included tumor necrosis factor-mediated signaling pathway, I-kappaB kinase/NF-kappaB signaling, and toll-like receptor signaling pathway (Figure 4G). These signaling pathway analyses highlighted the activation and inflammatory functions of the proinflammatory CD14^++^ MC.

### Specific Transcription Factors Predicted by SCENIC Regulated Activation of the Proinflammatory CD14^++^ MC

**FIGURE 5:**
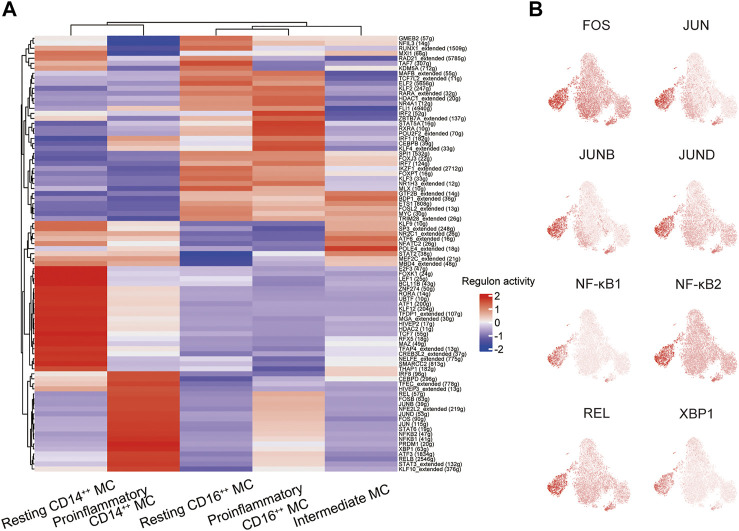
A gene regulatory network among the TFs predicted by SCENIC. **(A)** Heatmap of the AUC scores of transcription factors (TFs) in monocyte subsets, as estimated using SCENIC. **(B)** T-sne plots showing the regulon activity of the corresponding TFs (FOS, JUN, JUNB, JUND, NFKB1, NFKB2, REL, and XBP1).

We conducted single-cell regulatory network inference and clustering (SCENIC) analysis to evaluate the expression levels of transcription factors (TFs) in the four distinct monocyte subsets and explore potential TFs involved in the inflammatory responses. This computational method can predict critical regulators and their direct target genes (Van de Sande et al., 2020). We observed different SCENIC-predicted TFs expressed exclusively in specific monocyte clusters, including new and canonical transcription factors (Figure 5A). The proinflammatory CD14^++^ MC was enriched in inflammation-relevant TFs, such as FOS, JUN, JUNB, JUND, NF-κB1, NF-κB2, REL, and XBP1, compared to the other four subsets (Figures 5A,B). The abundance of these TFs may promote proinflammatory CD14^++^ MC in DME (Wagner and Eferl, 2005; Martinon et al., 2010; Sun, 2017). The motif enrichment of these TFs is mainly localized in the regions of the proinflammatory CD14^++^ MC (Figure 5B), consistent with the activity of inflammatory gene expression (Figures 4E,F). Through SCENIC analysis, we predicted candidate TFs of the proinflammatory CD14^++^ MC participating in the inflammatory process. Taken together, these results further demonstrated that the proinflammatory CD14^++^ MC was predominated in the pathological process of DME.

### Cell-Cell Communication Was Enhanced Among Monocytes and Other Immune Cells in DME

**FIGURE 6:**
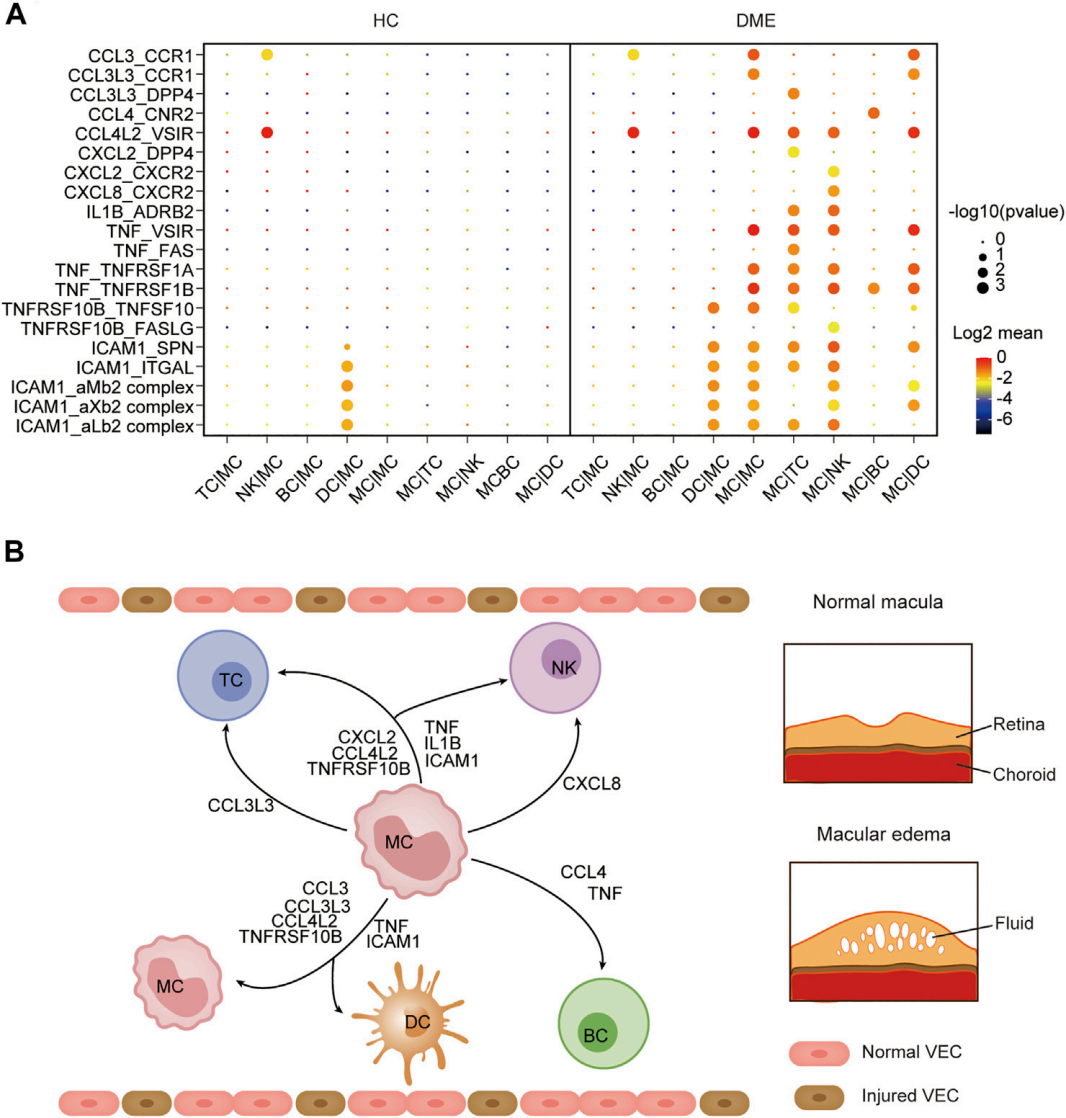
Cell-cell communication was enhanced among MC and other immune cells in DME. **(A)** Dot plot of the predicted interactions between monocytes and the indicated immune cell types in the HC and DME group. **(B)** Summary illustration depicting the potential cytokine-receptor interactions between MC and other types of peripheral immune cells in the DME.

The immune system is a complex network, and blood circulating immune cells can contact and influence each other by cell-cell interactions, which can be identified via scRNA-seq data analysis (Armingol et al., 2021). To understand how monocytes communicated with other four immune cell clusters in the DME patients, we applied CellPhoneDB (Efremova et al., 2020), a computational approach predicting cell-cell interactions by ligand-receptor partners analysis, to explore cellular behavior alterations of DME compared to HC (Figure 6A). We discovered that the interactions of eight chemokine, seven cytokine, and five adhesion molecule ligand-receptor pairs were significantly elevated in DME patients (Figure 6A). Notably, the interaction patterns of these ligand-receptor pairs were mostly from MC to other immune cell subsets, consistent with the high expression levels of inflammatory genes (Figure 2C, Figure 6A). Compared to the two groups, the CCL3-CCR1 and CCL3L3-CCR1 pair were limited to the MC-MC and MC-DC interaction, and the CCL3L3-DPP4 pair was only found in MC-TC (Figures 6A,B).

CCL4-CNR2 pair occurred only in MC-BC interaction (Figures 6A,B). In contrast, the CCL4L2-VSIR pair contributed to interactions of MC and other subsets except for BC (Figures 6A,B). MC can also recruit TC and NK through CXCL2 secretion. However, CXCL8 interacted uniquely with its receptor CXCR2 in NK (Figures 6A,B). The proinflammatory cytokine IL1B and receptor ADRB2 specially communicated MC and TC and NK (Figures 6A,B). The cytokine TNF contributed to a broad spectrum of cell communication through increased ligand-receptor pairs such as TNF-VSIR, TNF-FAS, TNF-TNFRSF1A, and TNF-TNFRSF1B (Figures 6A,B). The adhesion molecule ICAM1 played an extensive role in cell-cell interactions with its receptor such as SPN, ITGAL, aMb2, aXb2, and aLb2 complex (Figures 6A,B). Specifically, we found that MC required self-recruitment and activation through significantly increased cell interactions in DME patients (Figure 6A). These results confirmed that monocytes had a considerably enhanced propensity to initiate inflammation responses by secreting chemokines and cytokines. Taken together, these results predicted the possible molecular mechanisms underlying cell-cell communication in DME patients, further demonstrating the activation and proinflammatory signatures of MC.

## Discussion

It is increasingly recognized that chronic low-grade and sterile inflammation contributes to the pathogenesis of DR, from early phases to the vision-threatening advanced stages (Tang and Kern, 2011; Mesquida et al., 2019; Semeraro et al., 2019). Some studies had reported an increased adherence of leukocytes that contributes to physically capillary occlusion and microvascular damage by producing cytokines (Schroder et al., 1991; Ulbrich et al., 2003; Joussen et al., 2004). Despite decades of research, the immune mechanisms contributing to these processes in DME remain largely unresolved, and the identification of specific immune dysregulation is needed to develop new therapeutic strategies for DME. This study depicted the first single-cell immune atlas of peripheral blood in DME patients, allowing a precise understanding of inflammatory immune mechanisms. Compared to HC, a hyper-inflammatory response in DME was observed, which may explain why some DME patients had severe vision loss. In addition, we identified five major cell clusters with unique gene expression patterns and discovered that monocytes are the domain proinflammatory cells in DME patients. The monocytes were subdivided into four subsets, and their activation status, function signatures, and immune mechanisms were comprehensively described.

It has been demonstrated that monocytes are crucial participants in mediating chronic inflammatory diseases such as diabetes, atherosclerosis, and rheumatoid arthritis (Weber et al., 2008; Grossmann et al., 2015; Tabas and Lichtman, 2017; Cecchinato et al., 2018; Jordan et al., 2019; Zhang et al., 2019). Furthermore, accumulating evidence suggests that monocytes are involved in the pathogenesis of diabetic complications, including diabetic nephropathy and diabetic retinopathy (Nakajima et al., 2002; Theocharidis et al., 2020; Torres et al., 2020; Wan et al., 2020). In our study, monocytes in DME displayed unique differences and highly specialized functions compared to other immune cell subsets (Figures 2A,C,D). With DEG analysis of our transcriptional data, we observed that the inflammatory cytokines (*TNF*, *IL1B*, and *NFKBIA*) and chemokines (*CCL3*, *CCL4L2*, *CXCL2*, and *CXCL8*) are highly expressed in monocytes (Figures 2B,C). Compared to HC, monocytes showed higher expression of inflammatory genes in DME patients (Figures 2C,D), consistent with the high protein levels in vitreous and serum of DME patients reported in the previous studies (Ben-Mahmud et al., 2004; Demircan et al., 2006; Boss et al., 2017; Feng et al., 2018; Khaloo et al., 2020). Thus, it is reasonable to conclude that monocytes in DME exhibited an activated and proinflammatory status, thus enhancing the generation of chronic low-grade inflammation in the diabetic retina by releasing inflammatory cytokines and chemokines.

Human monocytes are traditionally subdivided into classical (CD14^++^ CD16^−^), nonclassical (CD14^dim^ CD16^++^), and intermediate monocytes (CD14^++^ CD16^+^), according to the relative expression of CD14 and CD16 (Ziegler-Heitbrock et al., 2010; Wong et al., 2011). Here, we discovered that CD14^++^ MC showed two distinct states based on the expression of inflammatory genes (Figures 3A,C–E). Therefore, we classified CD14^++^ MC into resting CD14^++^ MC and pro-inflammatory CD14^++^ MC (Figure 3A). We found that the upregulation of inflammatory genes expression, including inflammatory cytokines and chemokines, was largely focused on the proinflammatory CD14^++^ MC (Figures 4E,F). Moreover, our GO and pathway analysis indicated the proinflammatory CD14^++^ MC in DME was enriched with proangiogenic and proinflammatory pathways (Figure 4G). Previous studies have reported that VEGFA-VEGFR2 pathway contributed to neovascularization through regulating proliferation and sprouting of vascular endothelial cells, and also by increasing the permeability (Goel and Mercurio, 2013; Stitt et al., 2016). Hence, it is likely that the proinflammatory CD14^++^ MC may promote angiogenesis and macular edema with up-regulated VEGFA-VEGFR2 pathway. Corresponded to the high levels of inflammatory cytokines, the proinflammatory CD14^++^ MC was enriched in inflammatory cytokine-related pathways, such as signaling by interleukins, tumor necrosis factor-mediated signaling pathway, I-kappaB kinase/NF-kappaB signaling, toll-like receptor signaling pathway, and so on (Figure 4G). Consistent with the upregulation of chemokines, the proinflammatory CD14^++^ MC was characterized by highly upregulated myeloid leukocyte migration, chemotaxis, and differentiation pathways (Figure 4G). These data suggested the proinflammatory CD14^++^ MC was the predominant activated cell population in peripheral blood of DME patients.

Previous studies had reported AP-1 family TFs (FOS, FOSB, JUN, JUNB, and JUND), NF-κB family TFs (NF-κB1, NF-κB2, and REL), and XBP1 that participated in immune response, including immune cell activation, differentiation, and proinflammatory cytokines production (Wagner and Eferl, 2005; Martinon et al., 2010; Sun, 2017). Our DEG analysis indicated the proinflammatory CD14^++^ MC was uniquely characterized by higher gene expression of transcriptional factors (*FOS*, *FOSB,* and *JUNB*) (Figure 4D). Through SCENIC analysis, we predicted FOS, JUN, JUNB, JUND, NF-κB1, NF-κB2, REL, and XBP1 as key regulators directing inflammatory gene expression proinflammatory CD14^++^ MC (Figures 5A,B). These results may explain the transition from resting CD14^++^ MC to proinflammatory CD14^++^ MC in DME patients.

Interaction between monocytes and other immune cells may help understand the functional states of monocytes in DME. CellPhoneDB analysis predicted monocyte-centric ligand-receptor pairs and constructed interaction networks (Figures 6A,B). As a result, multiple inflammation-related ligand-receptor pairs’ expression was significantly increased in DME patients compared to HC (Figure 6A). Previous studies have noted that monocytes can sense environmental changes, migrate into lesions and differentiate into macrophages, playing a significant role in chronic inflammatory disease (Jakubzick et al., 2017). In our study, monocytes predominated in producing chemokines, cytokines, and adhesion molecules, which promoted interaction with other immune cells by different ligand-receptor pairs (Figure 6A). Early reports suggest increasing chemokines and cytokines results in immune cells recruitment to the diabetic retina and lead to immune dysregulation and retinal tissue damage (Rubsam et al., 2018).

Intravitreal anti-VEGF drugs are first-line treatments for DME, but a large fraction of patients didn’t show complete response (Antonetti et al., 2012). There is need to explore new therapeutics targeting VEGF-independent mechanisms, such as anti-inflammation drugs. Due to technical and ethical issues, we could only collect immune cells from patients’ peripheral blood samples but not directly from their retinal tissues. Nevertheless, we believe that a single-cell analysis on peripheral immune cells could provide new insight into the systemic control of diabetes that may reduce DME, possibly aided by anti-inflammatory agents targeting monocytes in peripheral blood. We revealed that the proinflammatory CD14^++^ MC in DME was uniquely characterized by the upregulation of inflammatory genes, including inflammatory cytokines (*TNF*, *IL1B*, *NFKBIA*, *DUSP2*, *NLRP3*, and *TNFAIP6*), chemokines (*CCL3*, *CCL3L1*, *CCL4L2*, *CXCL2*, and *CXCL8*), and transcriptional factors (*FOS*, *FOSB*, and *JUNB*). GO and pathway analysis of the DEGs demonstrated that the proinflammatory CD14^++^ monocytes upregulated inflammatory pathways including tumor necrosis factor-mediated signaling pathway, I-kappaB kinase/NF-kappaB signaling, and toll-like receptor signaling pathway. Further studies are needed to investigate the roles of these up-regulated inflammatory cytokines and chemokines and signaling pathways in the proinflammatory CD14^++^ MC for new drug development.

Our study was limited by the fact that we did not examine the entire immune landscape of DME patients, given that we only included PBMCs in our study, without including granulocytes and other immune cells. Further studies should be undertaken to investigate other cell populations associated with DME that were not fully addressed in the current paper, such as the NK cell sub-populations.

In conclusion, this study constructed the first immune landscape of DME patients with T2D and confirmed innate immune dysregulation in peripheral blood based on an unbiased scRNA-seq approach. With this high-resolution technology, one particular cell subset, the proinflammatory CD14^++^ MC, was identified to predominate in the pathogenesis of DME, providing a more comprehensive understanding of monocytes in human peripheral blood. Notably, we discovered that this subset in DME required a discriminative inflammatory gene expression signature, indicating its activated status and proinflammatory functions. Anti-inflammation treatments targeting this proinflammatory monocyte subset would be helpful for DME patients.

## Data Availability

The original contributions presented in the study are publicly available. This data can be found here: https://bigd.big.ac.cn/gsa-human/browse/HRA001139.
