# Supplementary material for: Immune Cell Landscape of Patients With Diabetic Macular Edema by Single-Cell RNA Analysis
Source: Front Pharmacol. 2021 Sep 14;12:754933. doi: 10.3389/fphar.2021.754933 (PMC8476792; doi:10.3389/fphar.2021.754933)
Supplement: Supplementary file 1 [file Table1.DOCX]

| **ident** | **Group** | **Gender** | **Age** | **Diabetes** | **Duration of diabetes (years)** | **Macular edema** |
| --- | --- | --- | --- | --- | --- | --- |
| **1** | HC | female | 64 | no | 0 | no |
| **2** | HC | male | 69 | no | 0 | no |
| **3** | HC | female | 28 | no | 0 | no |
| **4** | HC | male | 24 | no | 0 | no |
| **5** | DME | female | 78 | type 2 | 10 | yes |
| **6** | DME | female | 65 | type 2 | 16 | yes |
| **7** | DME | male | 50 | type 2 | 8 | yes |
| **8** | DME | female | 70 | type 2 | 10 | yes |

**Table S1 Characteristics of patients with DME.**
